# Expressional Changes in Cerebrovascular Receptors after Experimental Transient Forebrain Ischemia

**DOI:** 10.1371/journal.pone.0041852

**Published:** 2012-07-27

**Authors:** Sara Johansson, Gro Klitgaard Povlsen, Lars Edvinsson

**Affiliations:** 1 Department of Clinical Experimental Research, Glostrup Research Institute, Glostrup Hospital, Glostrup, Denmark; 2 Division of Experimental Vascular Research, Department of Clinical Sciences, Lund University, Lund, Sweden; The University of Hong Kong, Hong Kong

## Abstract

**Background:**

Global ischemic stroke is one of the most prominent consequences of cardiac arrest, since the diminished blood flow to the brain results in cell damage and sometimes permanently impaired neurological function. The post-arrest period is often characterised by cerebral hypoperfusion due to subacute hemodynamic disturbances, the pathophysiology of which are poorly understood. In two other types of stroke, focal ischemic stroke and subarachnoid hemorrhage, it has earlier been demonstrated that the expression of certain vasoconstrictor receptors is increased in cerebral arteries several days after the insult, a phenomenon that leads to increased contraction of cerebral arteries, reduced perfusion of the affected area and worsened ischemic damage. Based on these findings, the aim of the present study was to investigate if transient global cerebral ischemia is associated with upregulation of vasoconstrictive endothelin and 5-hydroxytryptamine receptors in cerebral arteries. Experimental transient forebrain ischemia of varying durations was induced in male wistar rats, followed by reperfusion for 48 hours. Neurological function was assessed daily by three different tests and cerebrovascular expression and contractile function of endothelin and 5-hydroxytryptamine receptors were evaluated by wire myography, immunohistochemistry and western blotting.

**Results:**

Transient forebrain ischemia induced neurological deficits as well as functional upregulation of vasoconstrictive ET_B_ and 5-HT_1B_ receptors in cerebral arteries supplying mid- and forebrain regions. No receptor upregulation was seen in arteries supplying the hindbrain. Immunohistochemical stainings and western blotting demonstrated expressional upregulation of these receptor subtypes in the mid- and forebrain arteries and confirmed that the receptors were located in the smooth muscle layer of the cerebral arteries.

**Conclusions:**

This study reveals a new pathophysiological aspect of global ischemic stroke, namely expressional upregulation of vasoconstrictor receptors in cerebral arteries two days after the insult, which might contribute to cerebral hypoperfusion and delayed neuronal damage after cardiac arrest.

## Introduction

Global ischemic stroke occurs when the blood supply to the entire brain or a large part of the brain is disrupted resulting in tissue deprivation of oxygen and glucose, which may give permanent brain damage. Several clinical conditions can give rise to global ischemic stroke but a frequent cause is cardiac arrest, which is a world-wide devastating health problem [1]–[3]. Even if systemic resuscitation and stable cardiac rhythm is achieved after a cardiac arrest, the post-arrest period often implies a period of cerebral no-reflow with multifocal ischemia followed by a short period of global cerebral hyperaemia, then a phase with prolonged cerebral hypoperfusion that may last for several days and give rise to cognitive and motor deficits [1], [2], [4]–[6]. Today, treatment options for normalization of post-arrest cerebral perfusion are still very poor [2], [3], [7]–[9].

Previous investigations have revealed that focal cerebral ischemia caused by transient middle cerebral artery (MCA) occlusion as well as global cerebral ischemia caused by subarachnoid hemorrhage (SAH) is associated with upregulation of vasocontractile endothelin type B (ET_B_) and 5-hydroxotryptamin type 1B (5-HT_1B_) receptors in cerebral arteries supplying the ischemic tissue [10]–[14]. This upregulation leads to increased arterial contractility and contributes to reduced cerebral perfusion and worsened ischemic brain damage. Furthermore, it has been shown that during cerebral ischemia levels of the vasoconstrictive substances endothelin-1 (ET-1) and 5-hydroxotryptamin (5-HT) are increased in plasma and cerebrospinal fluid [15], [16].

Despite different etiologies of cerebral ischemia, similarities exist in the resulting cellular pathophysiology. We hypothesise that upregulation of cerebrovascular vasoconstrictor receptors is a key phenomenon involved in many types of cerebral ischemia, after a direct cerebrovascular event or after a cardiac arrest. The purpose of this study was to investigate if transient forebrain ischemia induces changes in vasoconstrictor ET-1 and 5-HT receptors in cerebral arteries.

To induce global ischemic stroke in rats we employed a two-vessel occlusion and systemic hypotension model [17], in which both common carotid arteries are occluded for a period of time combined with lowering of the systemic blood pressure to allow minimal cerebral perfusion via the vertebral arteries. This model is frequently used to study the consequences of global ischemic stroke [3], [18]. The drop in cerebral blood flow (CBF) induced in this model is most pronounced in the forebrain and the anterior part of the lateral cortical lobe supplied by the anterior and middle cerebral arteries. In contrast, the hindbrain supplied by basilar and posterior cerebral arteries suffer little or no blood flow reduction [19], [20]. We hypothesise that this experimental transient forebrain ischemia gives rise to changes in vasoconstrictor ET_B_ and 5-HT_1B_ receptors in cerebral arteries supplying the ischemic area.

## Materials and Methods

### Ethics

All animal procedures were carried out strictly within national laws and guidelines and approved by the Danish Animal Experimentation Inspectorate (permit number: 2009-1679).

### Two-vessel Carotid Occlusion Model

Male wistar rats, weighing 270–350 g, were used for the experiments. Rats were fasted the night preceding the operation while allowing free access to tap water [21].

Transient forebrain ischemia was induced by a two-vessel carotid artery occlusion model [17]. After induction of anesthesia with 3.5% isoflurane (Abbott Laboratories) in N_2_O/O_2_ (70∶30) the rats were intubated and artificially ventilated with inhalation of 1.5–2% isoflurane in N_2_O/O_2_ (70∶30) during the surgical procedure. Blood samples were regularly withdrawn for blood gas analysis to check respiration (Radiometer, Copenhagen, Denmark). Catheters were inserted in the tail artery and vein for blood samples, infusions and monitoring of mean arterial blood pressure (MABP). The body and scull temperature were continuously monitored with a rectal and scull probe, respectively, and kept close to 37°C using external heating and cooling. Muscle relaxation was achieved with i.v. Norcuron (Shering-Plough) 0.2 mg/ml, bolus dose of 0.2 ml and thereafter 1.3 ml/h to maintain paralysis. A soft poly urethan catheter filled with 300 IU/ml heparin was inserted via the external jugular vein into the right atrium. Loose ligatures were placed around each of the common carotid arteries. After completion of the surgical procedure, rats received 0.5 ml heparin (100 IU/ml) i.v and were allowed 15–20 minutes of equilibration.

After equilibration, reversible ischemia was induced by lowering the MABP to 40 mm Hg by withdrawing blood through the jugular vein catheter, followed by clamping of both common carotid arteries. During the ischemia, the MABP was adjusted and held constant at 40 mmHg by regulating the amount of blood withdrawn in the jugular catheter. After the desired period of ischemia, normal blood pressure was restored by rapid reinfusion of blood and the carotid clamps were removed. A volume of 0.5 ml of 0.6 M sodium bicarbonate was injected intravenously to counteract systemic acidosis. When MABP had reached preischemic levels the jugular catheter was removed and incisions sutured. Animals were allowed to recover 15–20 minutes before discontinuation of isoflurane and extubation. Sham operated animals underwent the same surgical procedure with approximately the same surgery time, except for carotid clamping, lowering of MABP and sodium bicarbonate injection.

### Cerebral Blood Flow Measurements

To measure cortical cerebral blood flow (CBF) before, during and acutely after the ischemic insult and besides, a hole was drilled in the skull approximately 7 mm anteriorly of the bregma on the right side of the midline and a laser-Doppler probe connected to a blood flow meter was placed on the dura mater without perforation.

### Neurological Evaluation

#### Rotating pole test

This test evaluates the ability of the animals to traverse a horizontal pole, which was either steady or rotating at different speeds (3 or 10 rpm). At one end of the pole (45 mm in diameter and 150 cm in length) a cage with an entrance hole facing the pole and the floor covered with home cage bedding material, was placed. Rat performance was scored according to the following definitions: Score 1, the animal is unable to balance on the pole and fall off immediately; Score 2, the animal balances on the pole but has severe difficulty crossing the pole and moves <30 cm; Score 3, The animal embraces the pole with its paws and does not reach the end of the pole but does manage to move >30 cm; Score 4, the animal traverses the pole but embraces the pole with its paws and/or jumps with its hind legs; Score 5, the animal traverses the pole with normal posture but with >3 foot slips; Score 6, the animal traverses the pole perfectly with <3 foot slips [22].

#### Grip strength test

Forelimb grip strength (in grams) was evaluated using a grip strength meter [23]. The animal is placed on the platform with the forepaws inside the triangular grasping ring. Using one hand, the animal is grasped around the upper chest and the other hand on the base of the tail and then steadily pulled (≈1 in/sec) away from the forelimb grasping ring until the grip is broken and the trial is completed. Two successive readings were taken for each animal.

#### Muscle strength test

This test measures number of seconds the rat manages to hang upside-down in a grating, allowing the rat to grip the grid with all four paws before turning the grid. Maximum time score was set to 30 seconds and the average values of three trials were used.

### Harvest of Cerebral Arteries

48 hours following ischemia/sham-operation, rats were anesthetized with CO_2_ and decapitated. Brains were removed and chilled in ice-cold bicarbonate buffer solution. Under a dissection microscope, the basilar artery (BA), middle cerebral artery (MCA) and the anterior cerebral artery (ACA) were isolated.

### *In vitro* Pharmacology

A sensitive myograph (Danish Myograph Technology A/S) was used for recording the isometric tension in isolated cerebral arteries. Isolated vessels were cut into 1 mm long segments and mounted on two 40 µm in diameter stainless wires in the myograph. Measurements were recorded using a PowerLab unit and LabChart software (ADInstruments). The segments were immersed in temperature controlled buffer solution (37°C) of the following composition (mmol/L) NaCl 119, NaHCO_3_ 15, KCl 4.6, MgCl_2_ 1.2, NaH_2_PO_4_ 1.2, CaCl_2_ 1.5 and glucose 5.5. The buffer was continuously aerated with 5% CO_2_ to maintain a pH of 7.4. Vessel segments were stretched to initial pretension of 2 mN/mm and were allowed to equilibrate for 45 minutes. The vessels were then exposed to a solution of 63.5 mM K^+^ obtained by partial substitution of NaCl for KCl in the previously described buffer. Only vessels responding to the K^+^ buffer by contraction of at least 2.0 mN for BA and 0.7 mN for MCA and ACA were included in the study. Concentration-response curves were obtained by cumulative application of 5-carboxamidotryptamin (5-CT) (Sigma, St Louis, USA) in the concentration range 10^−12^ to 10^−5 ^M, endothelin-1 (ET-1) (AnsSpec, San jose, USA) and sarafotoxin 6c (S6c) (Sigma, St Louis, USA) in the concentration range 10^−14^ to 10^−7 ^M. The selective ET_B_ antagonist BQ788 (0.9*10^−6^ M, Sigma-Aldrich) and the 5-HT_1B/1D_ antagonist GR55562 (10^−6^ M, Tocris bioscience ) were used to confirm the involvement of these specific receptor subtypes in the contractile responses induced by ET-1 and 5-CT. When antagonists were used they were added 30 minutes before commencing application of cumulative doses of the relevant agonist.

### Immunohistochemistry

ACAs were dissected out, imbedded in Tissue TEK (Gibco) and frozen. They were sectioned into 10 µm thick slices using a cryostat (Leica Microsystems GmBH). After fixation in Stephanini’s fixative, sections were pre-incubated with phosphate-buffered solution (PBS) containing 5% donkey serum (Jackson ImmunoResearch Europe) and 1% bovine serum albumin (BSA). Primary antibodies used were sheep anti-ET_B_ (Alexis Biochemicals) diluted 1∶250, sheep anti-ET_A_ (Alexis Biochemicals) diluted 1∶100, rabbit anti-5-HT_1B_ (Abcam) diluted 1∶200, rabbit anti-5-HT_2A_ (Abcam) diluted 1∶200 and mouse anti-β-actin (Abcam) diluted 1∶500. Secondary antibodies used were DyLight 488-conjugated donkey anti-sheep antibody diluted 1∶200, DyLight 488-conjugated donkey anti-rabbit antibody 1∶200 and DyLight 549-conjugated donkey anti-mouse antibody 1∶200 (all from Jackson ImmunoResearch Europe). All antibodies were diluted in PBS containing 1% BSA, 0.25% Triton X-100 and primary antibodies dilution buffer also contained 2% donkey serum. Negative control slides went through the same staining procedure except that primary antibodies were not added. For detecting the secondary antibodies, appropriate laser wavelengths were used in a confocal microscope (Nikon D-eclips C1, Nikon Instruments). Images were analyzed using the software EZ-C1 3.70 FreeViewer, measuring the staining intensity in the smooth-muscle cell (β-actin positive) layer. All images were analyzed randomized in a blinded manner.

### Western Blot Analysis

BA, MCA and ACA from sham and 15 minutes induced ischemia rats were dissected out, cleared from extraneous connective tissue, depleted of blood and mechanically denuded for endothelium using a hair. MCA and ACA segments from the same rat were pooled together to a totally length of 17 mm and BA was pooled together from two different rats (of either ischemia or sham-operated group) to a length of 12 mm for a single n value, thereafter snap frozen on dry ice and kept at −80°C until preparation for immunoblot analysis.

Each sample was dissolved in 60 µl boiling cell extract denaturing LDS buffer (Termo Scientific) containing 50 mM DTT (Termo Scientific) and thereafter kept in −80°C for 1 hour. Cell lysates samples were then sonicated on ice for 3×15 pulses (30 outputs), centrifuged at 14 000 rpm (4°C) for 15 minutes and the supernatants were collected as final protein samples. Protein samples were preheated for 5 minutes and then separated on a 4–12% SDS Ready Gel Precast Gels (Expedeon, USA) for 70 minutes at 180 V and transferred to a PVDF membrane (GE Healthcare, Amersham Hybond-P) at 150 V for 70 minutes. The membranes were then blocked for unspecific binding for 1 hour at room temperature in TBS-T buffer (TBS +1% Tween20 (sigma) containing 5% BSA and thereafter incubated over night at 4°C with primary antibodies: Rabbit anti-ET_B_ (Alomone labs) diluted 1∶250, goat anti-5-HT_1B_ (Mybiosource) diluted 1∶250 or mouse anti-actin (Abcam) diluted 1∶15000. The day after, membranes were incubated for 1 hour with the HRP conjugated secondary antibodies: Donkey anti rabbit (GE Healthcare, UK) diluted 1∶20000, rabbit anti-goat (Thermo Scientific) diluted 1∶10000 or goat anti-mouse (Thermo Scientific) diluted 1∶20000. All antibodies were diluted in TBS-T buffer containing 5% BSA. Proteins were detected using the ECL Advanced Western Blot Kit, and visualized in a Fujifilm LAS-4000 Luminiscent Image Analyser and protein levels were normalized to the level of actin in each sample.

### Experimental Design, Statistics and Calculations

Eighty-seven male Wistar rats were used in the experiments. The rats were divided into five different groups, including sham (n = 28), 20 minutes of ischemia (n = 8), 15 minutes of ischemia (n = 35), 10 minutes of ischemia (n = 9) and 2 minutes of ischemia (n = 7). Data are presented as mean values ± standard error of the mean (SEM) and n refers to the number of rats.

Contractility curves were statistically analysed using 2-way ANOVA followed by Bonferroni’s posttest. Rotating pole test and the grip-strength test was analysed by 1-way ANOVA followed by Bonferroni’s multiple comparison test and the muscle-strength test was evaluated by two-tailed unpaired student’s t-test, comparing sham and 15 minutes ischemia group. Immunohistochemistry and western blot data were both analysed using two-tailed unpaired student’s t-test. p<0.05 was considered significant in all statistical analyses.

Vascular contractile responses are expressed as percentage of the contractile response to 63.5 mM K^+^. E_max_ represents the maximum contraction induced by an agonist and the pEC_50_ value refers to the negative logarithm of the concentration eliciting half maximal contraction. For biphasic responses, E_max(1)_ and pEC_50(1)_ describe the high-affinity phase and the E_max(2)_ and pEC_50(2)_ describe the low-affinity phase.

## Results

### Two-vessel Carotid Artery Occlusion Model

Rats were subjected to different durations of transient forebrain ischemia; 2, 10, 15 or 20 minutes, in order to investigate whether ischemia of different durations induced cerebrovascular receptor changes of different magnitude and in order to define the duration of ischemia needed to produce considerable neurological damage while keeping post-ischemic mortality at an ethically and practically feasible level.

The overall mortality rate among all rats was 9%. Four out of 35 rats died in the 15 minutes ischemia group and 4 rats in the 20 minutes ischemia group, the latter corresponding to 50% mortality in the 20 minutes ischemia group. The longest duration of ischemia used for further investigations was therefore 15 minutes. There were no significant differences between the groups in physiological parameters measured during the surgical procedure (Table 1).

**Table 1:** Physiological Parameters.

Group		MABP Pre-ischemia (mmHg)	MABP Ischemia (mmHg)	MABP Post-ischemia (mmHg)	pH Pre-ischemia	pH Post-ischemia	pCO_2_ Mean (kPa)	pO_2_ Mean (kPa)	Body T. Mean (°C)	Cranial T. Mean (°C)
**Sham**	104±9		–	–	7.4±0.1	–	4.1±0.6	16.8±2.2	36.5±0.1	–
**2 min**	108±12		42±6	114±10	7.4±0.1	7.4±0.1	4.7±0.2	19.9±2.4	36.9±0.6	36.9±0.5
**10 min**	113±17		40±3	127±19	7.4±0.1	7.3±0.1	4.2±0.3	18.4±2.6	36.8±0.6	36.7±0.5
**15 min**	107±12		42±4	122±13	7.4±0.1	7.3±0.1	4.7±0.7	17.6±2.4	36.7±0.5	36.7±0.5

Mean arterial blood pressure (MABP), pH, CO_2_ and O_2_ pressure (p) body temperature (T) and cranial temperature of animals subjected or not (sham) to 2, 10 or 15 minutes of transient forebrain ischemia. Values are means ± SEM, n = 7–17 rats in each group.

**Figure 1:**
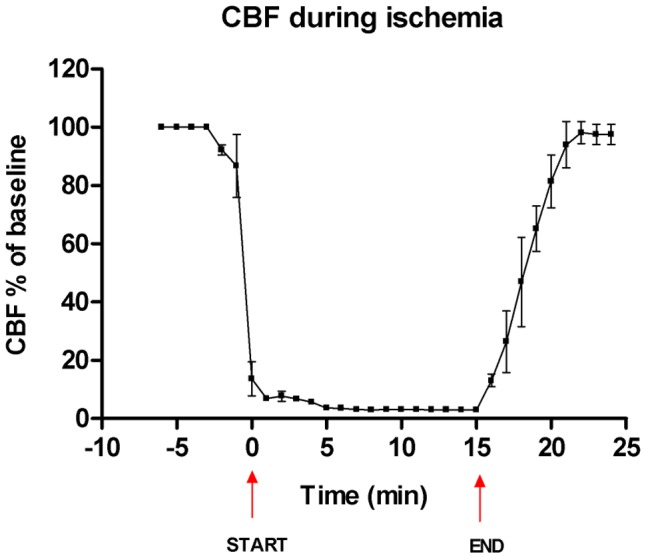
Cerebral blood flow during transient forebrain ischemia induction. Cortical cerebral blood flow (CBF) in the anterior part of the brain was measured before, during and after the 15 minutes transient forebrain ischemia with a laser-Doppler probe. The level of CBF before the ischemia (CBF baseline) was set to 100%. The two arrows depict the start and the end of the ischemic insult. Data are presented as means ± SEM (n = 2) in percentage of baseline CBF values.

To demonstrate that the used rat model was transient and that cerebral reperfusion was achieved after blood reinfusion and re-opening of the common carotid arteries, CBF was measured pre-, during and post-ischemia. Pre-ischemia CBF levels were set to 100% (baseline) and when the blood was withdrawn and the carotid arteries were clamped there was a rapid decrease of CBF to 5–6% of baseline. During ischemia, CBF was kept constant at 3–4% of baseline CBF. Immediately after reopening of the carotid arteries, CBF started to increase, and was increased to pre-ischemic levels (baseline) within 5 minutes after reopening of the carotid arteries (Figure 1).

### Neurological Outcome

In the group of rats subjected to 15 minutes ischemia, we observed changes in the general behaviour of the rats evident as hyperactivity and increasingly aggressive behaviour.

**Figure 2:**
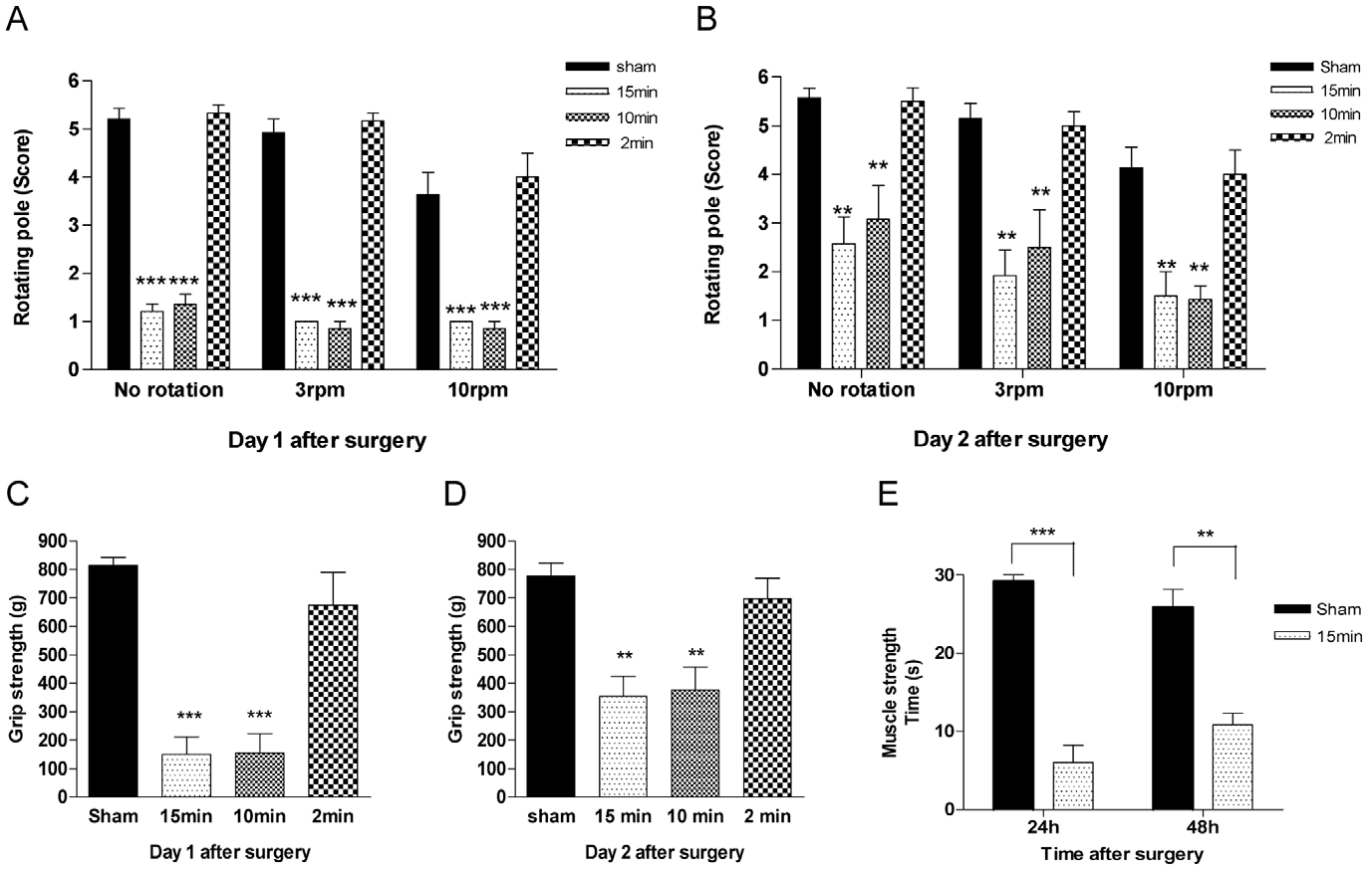
Neurological outcome. Bar graphs showing mean (± SEM) rotating pole scores (**A and B**) and grip strengths (**C and D**) for control-operated rats (sham) and ischemia-induced rats subjected to 15, 10 and 2 minutes of transient forebrain ischemia (15 min, 10 min, and 2 min, respectively). Asterisks indicate significant difference between sham and ischemia groups. (**A and B**) Rotating pole scores were assessed on day 1 (**A**) and day 2 (**B**) after surgery. The horizontal pole was static (no rotation) or rotating at 3 rpm or 10 rpm. Statistically significant differences were assessed by 1-way ANOVA followed by Bonferroni’s multiple comparison tests. For each rotation speed n = 3–7 rats in each group. (**C and D**) Grip strength was measured in gram (g) using a grip strength meter on day 1 (**C**) and day 2 (**D**) after surgery. Statistically significant differences were determined using 1-way ANOVA, n = 7–8 rats in each group. (**E**) Muscle strength was measured in seconds (s) rats managed to hang in a grid where highest score was set to 30 seconds. Statistical differences between sham and ischemia groups were determined using student’s t-test, n = 2–6 rats in each group. *p<0.05, **p<0.01 and ***p<0.001.

To examine the gross sensorimotor and neuromuscular functional deficits after transient forebrain ischemia, rats were tested at day 1 and day 2 after the ischemic insult using three different neurological tests; a rotating pole-, grip strength- and a muscle strength- test. In all three tests, animals subjected to 10 or 15 minutes of ischemia displayed neurological deficits reflected in significantly worse performance than sham-operated rats on both the first and second day after ischemia (Figure 2). In contrast, there was no significant difference between rats with 2 minutes induced ischemia and sham-operated rats in any of the tests (Figure 2). For all three tests, there was a non-significant tendency of the 10 and 15 minutes ischemia-induced rats to perform better on day 2 after the operation than on day 1.

### Contractile Responses of Cerebral Arteries

To investigate ET_B_ and 5-HT_1B_ vasoconstrictor receptors in cerebral arteries at a functional level, we determined the contractile responses to cumulative application of ET-1 (ET_A_ and ET_B_ receptor agonist) S6c (ET_B_ receptor-specific agonist) and 5-CT (5-HT_1_ receptor-selective agonist) in the following cerebral arteries; basilar artery (BA), middle cerebral artery (MCA) and anterior cerebral artery (ACA), using a wire myograph. Average K^+^-evoked responses as well as E_max_ and pEC_50_ values for agonist-induced contractions in the respective groups are presented in Table 2. K^+^-induced contractions did not differ significantly between the groups.

**Table 2:** Contractile Effects of 5-CT and ET-1 in Cerebral Arteries.

			Biphasic Curve				Sigmoidal Curve	
Group	n	K^+^ response	E_max(1)_ (%)	E_max(2)_ (%)	pEC_50(1)_ (−log M)	pEC_50(2)_ (−log M)	E_max_ (%)	pEC_50_ (−log M)
**5-CT BA**								
Sham	7	4.38±1.07	16.57±4.34	49.86±10.81	7.97±0.10	5.60±0.31		
15 min	8	3.87±1.40	24.50±5.31	50.87±5.74	8.20±0.25	5.35±0.30		
**5-CT MCA**								
Sham	8	1.81±0.58	25.63±5.05	63.88±12.28	7.94±0.35	5.36±0.35		
15 min	8	1.45±0.39	56.63±6.81	101.0±11.2	8.27±0.17	5.17±0.32		
10 min	5	1.79±0.62	70.0±10.6	118.8±13.1	8.21±0.19	5.39±0.38		
2 min	7	2.05±0.49	41.57±4.24	86.86±9.46	8.14±0.18	5.36±0.25		
**5-CT ACA**								
Sham	8	1.79±0.99	23.63±5.49	54.13±11.28	8.19±0.44	5.59±0.37		
15 min	8	1.84±0.76	54.0±12.2	89.63±16.02	8.31±0.38	5.53±0.54		
10 min	5	1.74±0.43	39.00±7.49	103.6±12.9	8.10±0.36	5.56±0.20		
2 min	7	1.87±0.57	42.71±9.66	93.86±11.52	8.05±0.36	5.76±0.23		
**ET-1 BA**								
Sham	7	4.38±1.07					90.86±8.88	9.23±0.16
15 min	8	3.87±1.40					87.75±11.52	9.36±0.37
**ET-1 MCA**								
Sham	8	1.67±0.59					148.0±8.8	8.73±0.11
15 min	7	1.46±0.44	44.86±9.22	156.4±26.6	11.88±0.77	8.95±0.24		
10 min	5	1.79±0.62					166.8±19.7	8.77±0.10
2 min	7	2.05±0.49					115.4±16.9	8.83±0.23
**ET-1 ACA**								
Sham	8	1.82±0.99					88.25±13.60	9.51±0.19
15 min	7	1.85±0.77	43.57±7.61	140.7±13.2	11.78±0.51	9.14±0.17		
10 min	5	1.74±0.43	44.00±9.57	147.8±17.9	11.41±0.48	9.04±0.22		
2 min	7	1.87±0.57					113.4±11.0	9.21±0.17

Pharmacological parameters for contractile responses to endothelin-1 (ET-1) and 5-carboxamidotryptamine (5-CT) in basilar arteries (BA), middle cerebral arteries (MCA) or arterial cerebral arteries (ACA) subjected to control (sham) operated or to 2, 10 or 15 minutes of transient forebrain ischemia. Contractile responses were characterized by maximum contractile response (E_max_) values, expressed as percentage of 63 mM K+ induced contraction (K^+^ response), and values of the negative logarithm of the molar concentration that produces half maximum contraction (pEC_50_). For biphasic concentration-contraction curves, E_max_ and pEC_50_ values for each of the two phases are provided. Values are means ± SEM, n =  numbers of rats.

#### Contractile responses to ET-1

**Figure 3:**
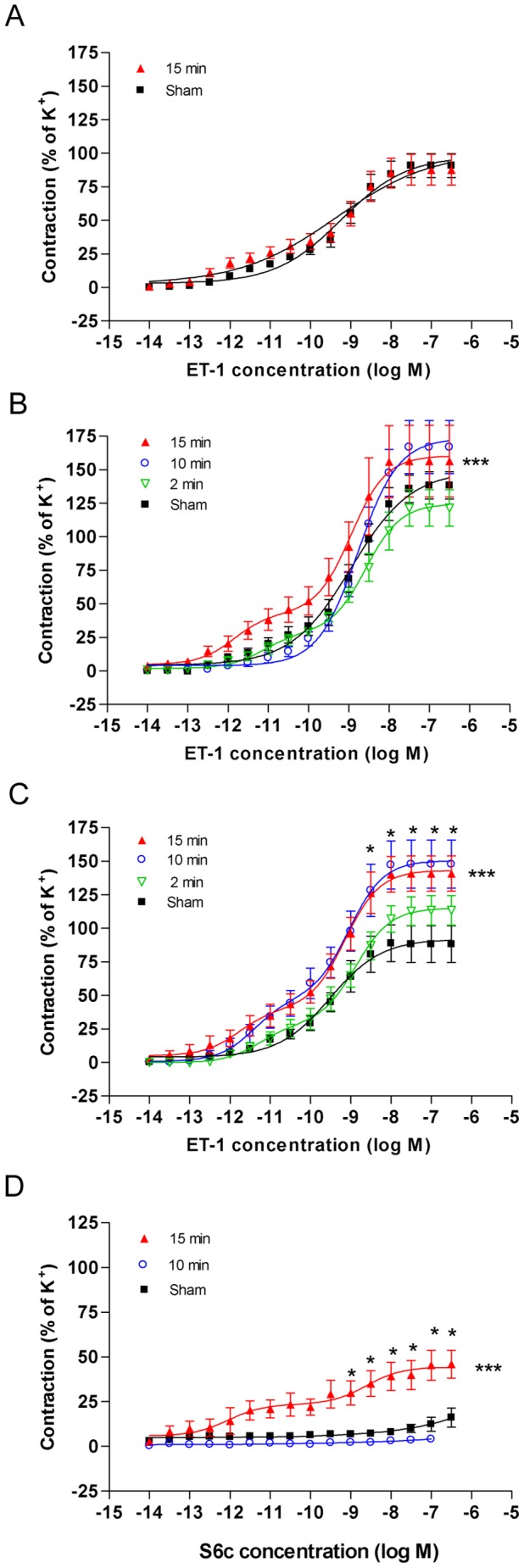
Contractile function of endothelin receptors in cerebral arteries. Graphs showing concentration-contraction curves elicited by the cumulative application of endothelin-1 (ET-1) to basilar artery (BA) (**A**), middle cerebral artery (MCA) (**B**) and anterior cerebral artery (ACA) (**C**) segments from control-operated rats (sham) and rats subjected to 15, 10 or 2 minutes of transient forebrain ischemia (15 min, 10 min, and 2 min, respectively). (**D**) Shows contractile responses to the ET_B_-specific agonist sarafatoxin 6c (S6c) in ACA segments from sham-operated rats and rats subjected to 10 or 15 minutes of transient forebrain ischemia. Values are expressed as means ± SEM in percentage of contractions evoked by 63 mM of K+. Significant differences between sham and 15 minutes ischemia are determined by 2-way ANOVA followed by Bonferroni’s posttest indicated by the asterisks on the right side respective above the curves.

Contractile responses to ET-1 were enhanced in MCAs and ACAs from 15 min induced ischemia rats (Figure 3B and C). The enhancement was observed as leftward shifts of the ET-1 concentration-contraction curves with significantly elevated E_max(2)_ values and transition into biphasic curve as opposed to sham-operated rats for which sigmoidal curves were obtained. Thus, in ACA of 15 min of induced ischemia the maximal response (E_max(2)_) to ET-1 were increased to 159±14% of sham operated rats and in addition, an E_max(1)_ for the first phase of the curve of 204±49% of the corresponding responses in sham-operated rats appeared (Figure 3C). In contrast, in BA the ET-1 responses were unchanged by the ischemia (Figure 3A).

For rats with 10 minutes induced ischemia, the ET-1 response in the ACA was of biphasic curve shape and upregulated to the same extent as in ACAs from 15 minutes ischemia-induced rats, whereas MCAs from rats with 10 minutes ischemia showed only a partial upregulation of the ET-1 response and a sigmoidal curve shape. For rats with 2 minutes induced ischemia, normal sigmoidal curves were obtained in both MCA and ACA, and ET-1 responses in the MCA were partially upregulated, whereas ET-1 responses in ACAs from rats with 2 minutes of ischemia were unchanged as compared to sham-operated rats (Figure 3B and C).

As earlier demonstrated in cerebral arteries after subarachnoid hemorrhage, the biphasic ET-1 concentration-contraction curves of MCAs and ACA from rats with 15 minutes induced ischemia indicate the presence of two contractile ET receptor subtypes, ET_B_ and ET_A_, where the first phase reflects contraction mediated by ET_B_ receptors and the second phase is dominated by ET_A_-mediated contraction, whereas the sigmoidal curves seen in sham-operated rats reflects purely ET_A_-mediated contraction [10]. This was confirmed in experiments using the ET_B_ receptor specific antagonist BQ788, which was found to diminish the first phase of the ET-1 concentration-contraction curves MCA from rats with 15 minutes induced ischemia (data not shown).

#### Contractile responses to S6c

To further confirm the appearance of contractile ET_B_ receptors in cerebral arteries from rats subjected to transient forebrain ischemia, the specific ET_B_ receptor agonist S6c was used. S6c yielded no contractile response in vessels from sham-operated rats or rats with 10 minutes of induced ischemia, but yielded a significant contractile response in ACA from 15 minutes induced ischemia rats, indicating the occurrence of contractile ET_B_ receptors in these arteries after transient forebrain ischemia (Figure 3D).

#### Contractile responses to 5-CT

**Figure 4:**
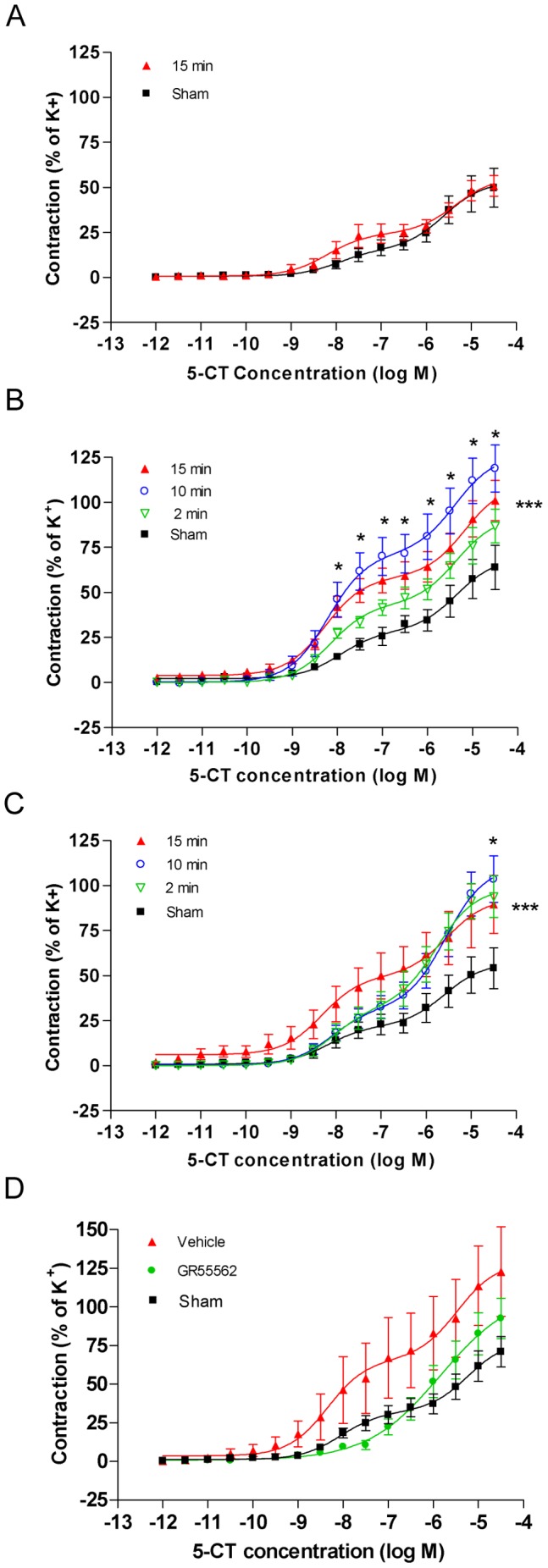
Contractile function of 5-HT_1B_ receptors in cerebral arteries. Graphs showing concentration-contraction curves elicited by the cumulative application of 5-carboxamidotryptamine (5-CT) to basilar artery (BA) (**A**), middle cerebral artery (MCA) (**B**) and anterior cerebral artery (ACA) (**C**) segments from control-operated rats (sham) and rats subjected to 15, 10 or 2 minutes of transient forebrain ischemia (15 min, 10 min, and 2 min, respectively). (**D**) Shows contractile response to 5-CT in MCA segments from sham-operated rats and rats subjected to 15 minutes of ischemia, in the presence and absence of the selective 5-hydroxotryptamin type 1B receptor (5-HT_1B_) antagonist GR55562. Values are expressed as means ± SEM in percentage of contractions evoked by 63 mM of K+. Significant differences between sham and 15 minutes ischemia are determined by 2-way ANOVA followed by Bonferroni’s posttest indicated by the asterisks on the right side respective above the curves.

5-CT elicited biphasic concentration-contraction curves in both sham-operated and ischemia-induced rats in all three types of cerebral arteries. These biphasic curves are due to the presence of two 5-HT receptor subtypes, 5-HT_1B_ and 5-HT_2A_, where the first high-affinity phase reflects stimulation of 5-HT_1B_ receptors, and the second low-affinity phase is dominated by 5-HT_2A_-mediated contraction [11], [24]. In both MCA and ACA from rats with 15 minutes of induced ischemia, significantly elevated contractile responses to 5-CT were observed as compared with sham-operated rats (Figure 4B and C). Thus, the maximal response to 5-CT in ACA after 15 minutes of induced ischemia was increased to 165±30% of the response in sham-operated rats (Figure 4C). In contrast, in BA the 5-CT response was unchanged by the ischemia (Figure 4A).

For rats with 10 minutes induced ischemia, 5-CT response in the MCA were increased to the same extent as in the 15 minutes ischemia-induced rats, whereas in the ACA 10 minutes of ischemia gave rise to a partial upregulation of the 5-CT response as compared to 15 minutes of ischemia (Figure 4B and C). For rats with 2 minutes of induced ischemia, 5-CT responses in both MCA and ACA were partially upregulated compared to the 15 minutes ischemia (Figure 4B and C). In order to investigate the specific involvement of the 5-HT_1B_ receptor in the enhanced responses to 5-CT in ischemic rats, 5-CT concentration-contraction curves in the presence of the 5-HT_1B_ selective antagonist GR55562 were obtained in MCAs from rats with 15 minutes induced ischemia. This inhibitor attenuated first phase of the enhanced 5-CT-induced concentration-contraction curves in the ischemic rats, indicating that the enhanced responses in these rats are due to upregulation of contractile 5-HT_1B_ receptors (Figure 4D).

In summary, experimental forebrain ischemia induced enhanced contractile response mediated by the receptor subtypes ET_B_ and 5-HT_1B_ in MCA and ACA, but not in BA.

### Expression of Vasoconstrictor Receptors in Cerebral Arteries

#### Immunohistochemistry analysis

**Figure 5:**
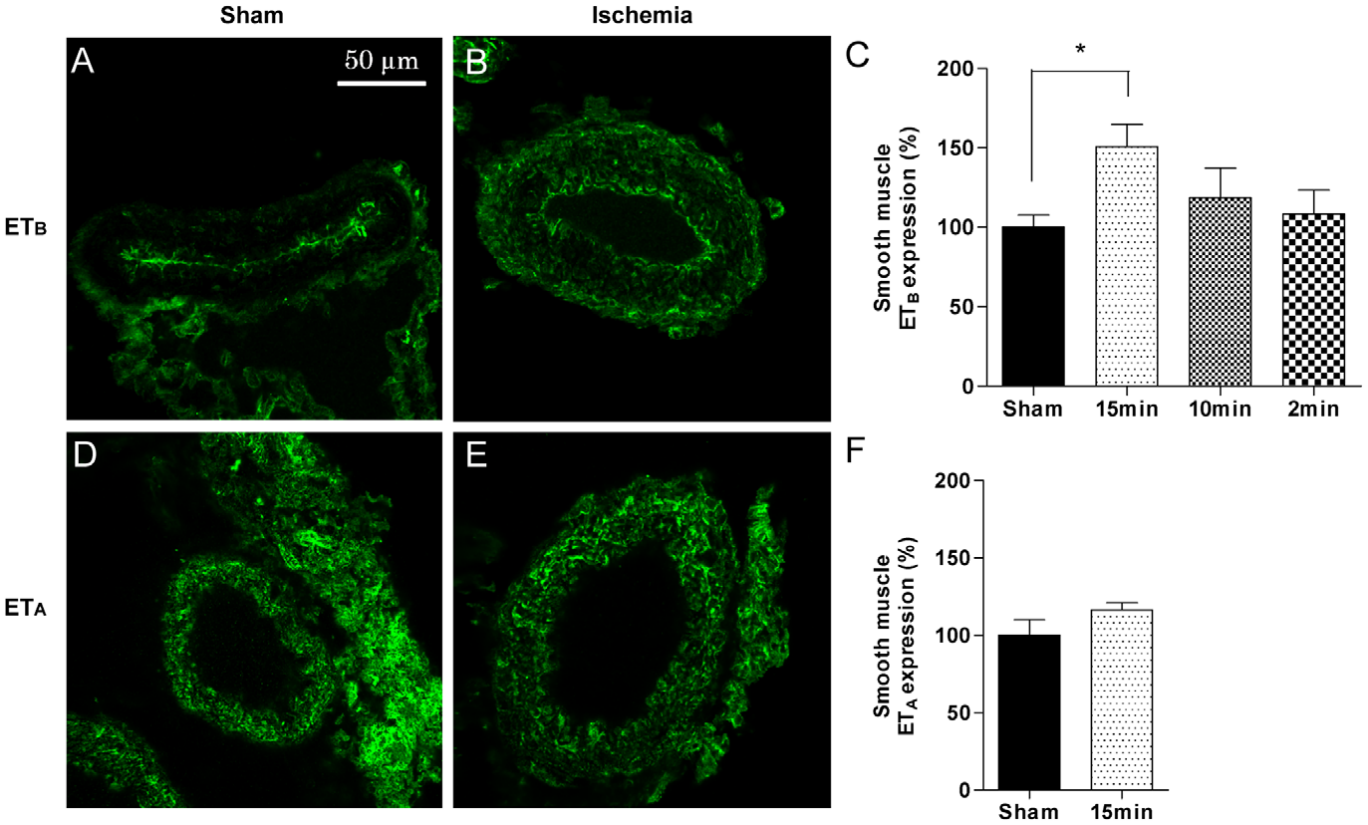
Endothelin receptor immunohistochemistry. Photomicrographs showing immunohistochemical stainings of anterior cerebral artery (ACA) sections with antibodies against endothelin type B (ET_B_) (**A and B**) or endothelin typ A (ET_A_) (**D and E**) receptors. Bar graphs show quantifications of staining intensities for ET_B_ (**C**) and ET_A_ (**F**) receptors. Two sections from each of 6 to 11 animals were analyzed. Data are presented as mean 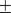 SEM in percentages of the mean staining intensity in control-operated (sham) animals. Significant differences between sham-operated and 15 minutes ischemia rats were determined using student´s t-test. *p< 0.05.

**Figure 6:**
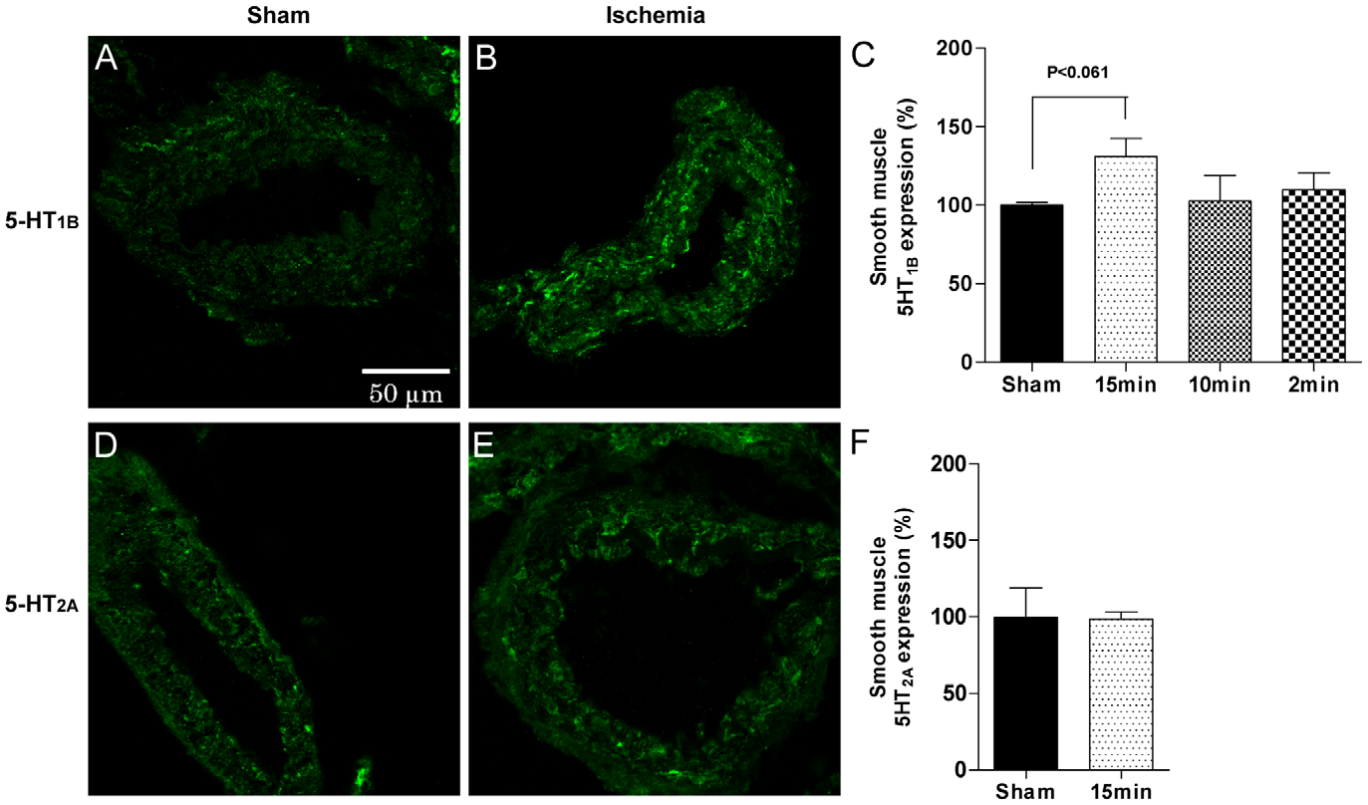
5-HT receptor immunohistochemitry. Photomicrographs showing immunohistochemical stainings of anterior cerebral artery (ACA) sections with antibodies against 5-hydroxotryptamin type 1B (5-HT_1B_) (**A and B**) or 5-hydroxotryptamin type 2A (5-HT_2A_) (**D and E**) receptors. Bar graphs show quantifications of staining intensities for 5-HT_1B_ (**C**) and 5-HT_2A_ (**F**) receptors. Two sections from each of 6 to 12 animals were analyzed. Data are presented as mean ± SEM in percentages of the mean staining intensity in control-operated (sham) animals. Significant differences between sham-operated and 15 minutes ischemia rats were determined using student’s t-test.

To investigate the expression of vasoconstrictor receptors in the smooth muscle of cerebral arteries at protein levels, ACAs were costained with antibodies against smooth muscle actin and_,_ ET_B_, ET_A_, 5-HT_1B_ or 5-HT_2A_ receptors using immunohistochemistry. In ACAs from 15 minutes induced ischemia rats, ET_B_ receptor protein was expressed in the smooth muscle cells and the staining intensity was significantly increased compared with ACAs from sham-operated rats (Figure 5A, B and C). The 5-HT_1B_ receptor was expressed in the smooth muscle cells in ACAs from 15 minutes ischemia rats, with a non-significant tendency to increased staining intensity compared to ACAs from sham-operated rats (Figure 6A, B and C). In contrast, the 5-HT_2A_ and ET_A_ receptor proteins showed no differences in staining intensity between ischemia-induced and sham-operated rats (Figure 5D, E, F and Figure 6D, E, F, respectively).

#### Western blot analysis

**Figure 7:**
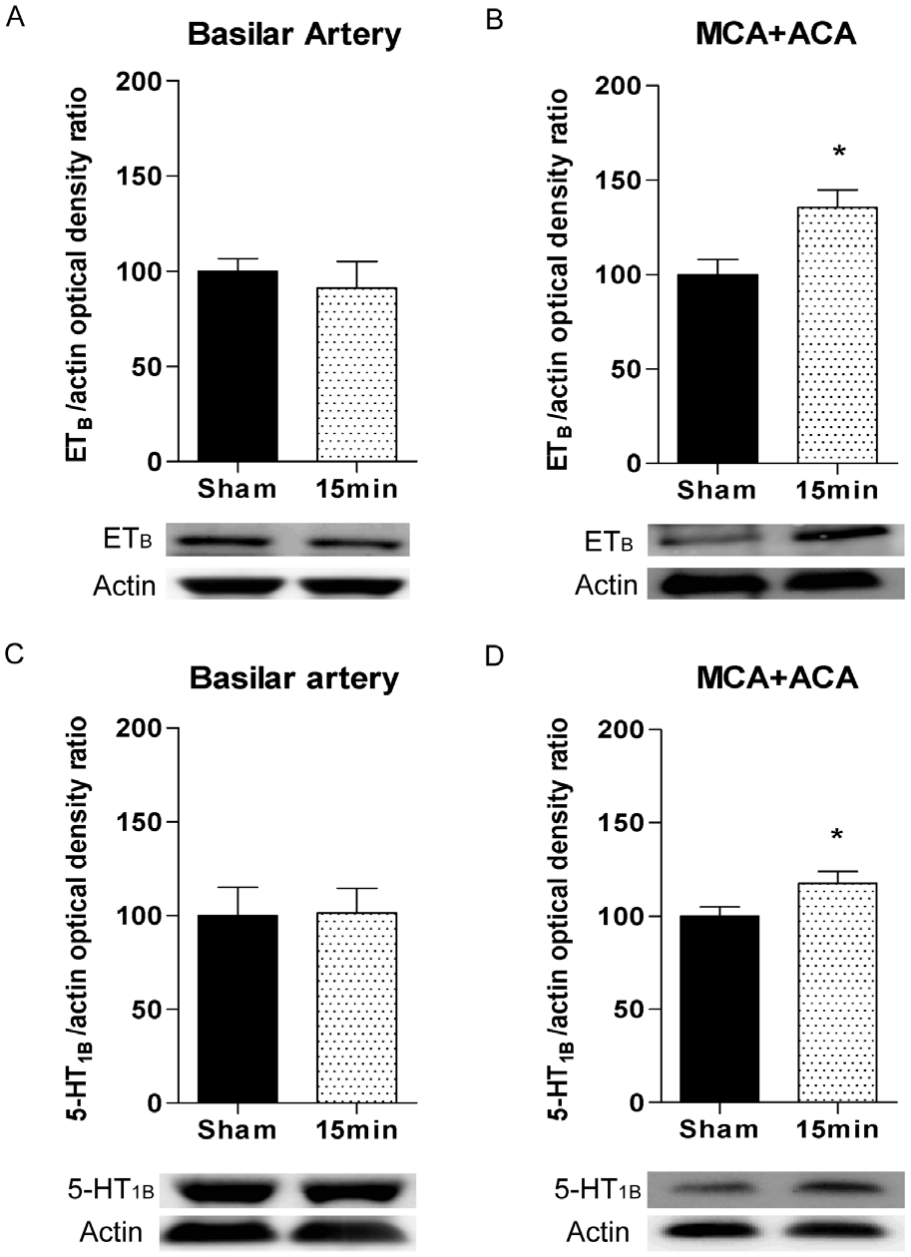
ET_B_ and 5-HT_1B_ receptor expression determined by western blotting. Representative western blots and band intensity quantifications showing endothelin type B (ET_B_) protein expression in basilar artery (BA) (**A**) and in pooled middle cerebral artery (MCA) and anterior cerebral artery (ACA) tissue (**B**), and 5-hydroxotryptamin type 1B (5-HT_1B_) protein expression in BA (**C**) and in pooled MCA and ACA tissue (**D**) from control-operated (sham) rats and rats subjected to 15 minutes of transient forebrain ischemia. The band recognised by the ET_B_ receptor antibody was approximately 48 kDa and the band recognised by the 5-HT1B receptor antibody was approximately 41 kDa. Actin was used as a loading control. Data are expressed as mean ± SEM percentages of the mean band intensity in sham-operated animals and student’s t-test was used for statistical comparison. n = 5–13. *p<0.05.

The protein levels of ET_B_ and 5-HT_1B_ receptors in BA, MCA and ACA from sham and 15 minutes ischemia induced rats were evaluated by Western blotting. Immunoblotting with antibodies against the ET_B_ receptor yielded two bands of app. 48 kDa and 42 kDa, respectively. When an ET_B_ receptor epitope mimetic blocking peptide was used the ET_B_ receptor band of 48 kDa was not observed (data not shown) confirming that this band represents the ET_B_ receptor. In pooled MCAs and ACAs from 15 minutes ischemia rats, the ET_B_ receptor band (48 kDa) showed significantly increased intensity compared to MCAs and ACAs from sham-operated rats when normalized to the level of actin (loading control) (Figure 7B). The 5-HT_1B_ receptor was visualized as a single band of app. 41 kDa, and intensity quantification and normalization to actin levels of 5-HT_1B_ receptor immunoblots showed significantly increased 5-HT_1B_ receptor expression in MCAs and ACAs from ischemia rats compared to MCAs and ACAs from sham-operated rats (Figure 7D). In contrast, the expression of 5-HT_1B_ and ET_B_ receptors in BA was unchanged between ischemia induced and sham-operated rats (Figure 7A and C).

**Figure 8:**
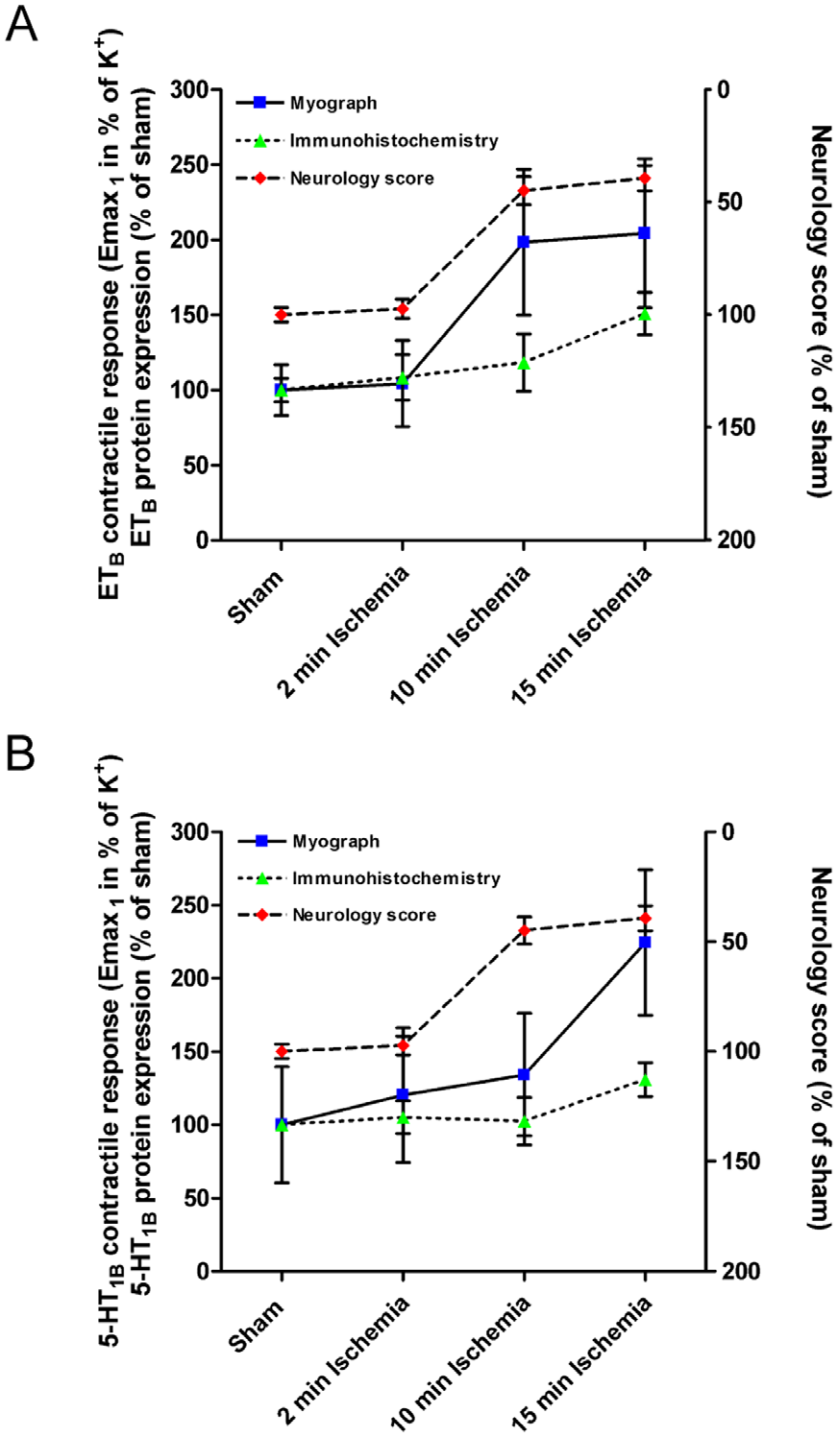
Summary of data for neurological function, contractile function and protein expression levels of cerebrovascular ET_B_ and 5-HT_1B_ receptors as a function of the duration of the ischemic insult. Data for endothelin type B (ET_B_) and 5-hydroxotryptamin type 1B (5-HT_1B_) receptor-mediated contractile function represent E_max_ values for the first phase (E_max-1_) of the biphasic concentration-contraction curves for anterior cerebral arteries (ACA) stimulated with endothelin-1 (ET-1) and 5-carboxamidotryptamine (5-CT), respectively. Data for ET_B_ and 5-HT_1B_ protein expression levels represent receptor subtype-specific immunohistochemical staining intensities in ACAs. Neurology score data represents pooled data from grip strength and rotating pole. All data are values for ischemia-induced rats (2, 10 or 15 minutes) terminated 48 hours after ischemia in percentages of corresponding values for control-operated (sham) rats. All data are expressed as mean ± SEM.

Figure 8 summarises data for ET_B_ and 5-HT_1B_ receptor contractile functionality, receptor protein expression and neurological outcome as a function of the duration of the ischemic insult.

## Discussion

The most critical consequence of cardiorespiratory arrest is its effects on the brain; the acute global ischemic stroke combined with cerebral hypoperfusion and delayed neuronal cell death in the post-arrest period often leave the patient with significant neuronal damage and long-term neurological deficits [5]–[7], [10]. In the present study, we demonstrate for the first time that cerebral arteries undergo changes in their expression of vasoconstrictor receptors after experimental forebrain ischemia, shifting their phenotype towards increased contractility. These increased cerebrovascular constrictor receptor levels may contribute to cerebral hypoperfusion and delayed brain damage after global ischemic stroke, which are poorly understood pathophysiological phenomena.

The data in the present study show that ET_B_ and 5-HT_1B_ receptors are upregulated in MCA and ACA smooth muscles at 48 hours after experimental forebrain ischemia, and that the degree of receptor upregulation depends on the duration of the ischemic insult. Thus, MCA and ACA from rats with 15 minutes of induced ischemia showed significant enhancement of contractile responses mediated by 5-HT_1B_ and ET_B_ receptors, whereas elevated contractile responses found in MCA and ACA after 10 minutes of ischemia were not as pronounced. Even 2 minutes of experimental forebrain ischemia induced a weak partial enhancement of ET_B_ and 5-HT_1B_ receptor contractile function in MCAs and ACA, indicating a very sensitive response in the cerebral vasculature to even very short ischemic incidences. Immunohistochemical stainings showed that 15 minutes of ischemia caused a significant upregulation of ET_B_ and 5-HT_1B_ protein expression levels, a finding that was confirmed by western blotting. However, the partial enhancement of ET_B_- and 5-HT_1B_-mediated contractile responses after 2 and 10 minutes of ischemia was not reflected in detectably increased protein levels of these receptors as determined by immunohistochemistry. This indicates that the enhanced contractile responses after 2 and 10 min ischemia are due to mere functional, not expressional, changes in the receptors, whereas the more profoundly enhanced contractile responses in rats with 15 minutes induced ischemia reflects expressional upregulation of the receptors.

The neurological deficits in the rats also depend on the duration of the forebrain ischemia. 10 and 15 minutes of ischemia induced significant neurological damage, whereas 2 minutes of ischemia was not enough to affect neurological outcome. However, 2 minutes of experimental transient forebrain ischemia has previously been shown to be sufficient to give rise to delayed neuronal damage in vulnerable brain areas [25]. Thus, damage has been detected at 7 days after induction of 2 minutes transient forebrain ischemia in hippocampal regions CA1 and CA4. After 4 minutes of induced ischemia the neuronal cell damage was more consistent and after longer ischemic durations such as 8–13 minutes, the damage was even more pronounced and more widely distributed [25], [26]. Depending on the duration of the ischemia, neuronal cell death begins to occur from 2 to 4 days after the insult and reaches its maximum within 1–2 weeks [26], [27]. Since the pattern of cerebrovascular receptor upregulation demonstrated in the present study correlates well with this earlier reported development of delayed neuronal damage, we hypothesise that the upregulation of vasoconstrictor receptors in cerebral arteries play a role in the development of delayed neuronal damage after forebrain ischemia by increasing the contractility of affected cerebral arteries thereby compromising optimal blood perfusion of brain areas threatened by the ischemic damage.

Hence, both the degree of functional and expressional receptor upregulation and the neurological deficits depend on the duration of the ischemic insult as summarised in Figure 8. However, whether the upregulation of vasoconstrictor receptors is directly involved in determination of the neurological outcome remains speculative and cannot be concluded on the basis of the data presented here.

The acute time-course of cerebral reperfusion after reopening of the carotid arteries and normalization of the systemic blood pressure in the here employed model has been characterized in detail earlier [28]. Local CBF in the forebrain regions during the ischemia is less than 5% of control levels CBF before the ischemia) and after 60 minutes of recirculation values are around 50% of control [28] indicating that the rats suffer a post-ischemic hypoperfusion phase lasting for at least 60 minutes [29]. However, whether the model is associated with any longer-term change in cerebral perfusion has not been addressed yet, although as mentioned earlier, it is known that the brain damage in this model is delayed and is not fully developed until approximately a week after the insult. The occurrence of periods of reduced cerebral perfusion during the first week after the insult and the possible role of cerebrovascular receptor changes in this model, is a novel aspect with clear relevance to the clinical problem of cerebral hypoperfusion after global ischemic stroke, which deserves further investigation in future studies.

Interestingly, we found no upregulation of neither ET_B_ nor 5-HT_1B_ receptors in the BA after 15 minutes ischemia, indicating a selective receptor upregulation in arteries supplying the forebrain. Hence, upregulation of vasoconstrictor receptors only takes place in cerebral arteries that are subjected to a considerable drop in blood flow during the ischemic insult and the pattern of vasoconstrictor receptor upregulation follows the anatomical distribution of the resulting brain damage. This is in accordance with previous findings from rat models of focal ischemic stroke, induced by 2 hours MCA occlusion followed by reperfusion. Here, contractile receptor upregulation exclusively occurs in the occluded MCA and not in the contralateral MCA [14], [30]. Accordingly, in the case of a rat SAH model characterised by a dramatic acute drop in global CBF followed by a delayed global cerebral ischemia 2–3 days after the SAH, similar vasoconstrictor receptor upregulation occurs in all explored cerebral arteries 48 hours after induction of SAH [22], [31]. On this basis, we speculate that the receptor changes are initiated by the initial drop in blood flow in the affected arteries. This is supported by recent data from a permanent distal MCA occlusion rat model where upregulation of contractile ET_B_ receptors was observed only in segments located downstream of the occlusion. In this model, almost no ischemic damage was seen in the tissue surrounding the MCA, either upstream or downstream from the occlusion, indicating that the receptor upregulations are a consequence of the local reduction in arterial wall tension after the occlusion rather than a result of brain tissue ischemia [32].

There is an urgent need for novel pharmacological means to treat the consequences of ischemic stroke. Neuroprotective agents have been tested, mainly in focal ischemic stroke, but with poor outcomes in major clinical trials [33]. The present study reveals a novel pathophysiological consequence of experimental forebrain ischemia, namely the upregulation of cerebrovascular vasoconstrictor receptors. In models of focal ischemic stroke and SAH, inhibition of intracellular signalling pathways mediating the upregulation of both 5-HT_1B_ and ET_B_ receptors has been shown to diminish cerebrovascular constriction and cerebral ischemia after the insults [13], [22], [34]. In contrast, inhibition of endothelin receptors alone with specific receptor antagonists have produced mainly disappointing results [35], [36]. On this basis, we speculate that the here reported changes in vasoconstrictor receptor expressions following experimental forebrain ischemia could be a novel target for future pharmacological means for attenuation of cerebral hypoperfusion and ischemic damage following cardiac arrest and global ischemic stroke.
